# Clinical Significance of Antineutrophil Cytoplasmic Antibody Positivity in Patients Infected with SARS-CoV-2

**DOI:** 10.3390/jcm11144152

**Published:** 2022-07-17

**Authors:** Lucy Eunju Lee, Wooyong Jeong, Yong-Beom Park, Su Jin Jeong, Sang-Won Lee

**Affiliations:** 1 Division of Rheumatology, Department of Internal Medicine, Yonsei University College of Medicine, Seoul 03722, Korea; elee@dumc.or.kr (L.E.L.); yongbpark@yuhs.ac (Y.-B.P.); 2 Division of Rheumatology, Department of Internal Medicine, Dongguk University Ilsan Hospital, Goyang 10326, Korea; 3 Department of Infectious Diseases, National Health Insurance Service Ilsan Hospital, Goyang 10444, Korea; jwym777@yuhs.ac; 4 Institute for Immunology and Immunological Diseases, Yonsei University College of Medicine, Seoul 03722, Korea; 5 Division of Infectious Diseases, Department of Internal Medicine, Yonsei University College of Medicine, Seoul 03722, Korea

**Keywords:** antineutrophil cytoplasmic antibody, SARS-CoV-2, significance, vasculitis

## Abstract

Objectives: To investigate the rate of antineutrophil cytoplasmic antibody (ANCA) positivity and its clinical significance in patients infected with severe acute respiratory syndrome coronavirus 2 (SARS-CoV-2). Methods: This study included 178 patients infected with SARS-CoV-2 who were enrolled in a cohort at a single centre. Myeloperoxidase (MPO)-ANCA and proteinase 3 (PR3)-ANCA levels in stored blood sera were measured using immunoassay kits. Mortality, mechanical ventilator care, and severe infection were assessed as three poor outcomes. The 2022 American College of Rheumatology and the European Alliance of Associations for Rheumatology (ACR/EULAR) classification criteria for the three subtypes of AAV were applied only to patients who had MPO-ANCA or PR3-ANCA among study subjects. Results: The detection rate of ANCA positivity was 18.5%. MPO-ANCA and PR3-ANCA were found in 22 (12.4%) and 14 (7.9%) patients, respectively. However, neither MPO-ANCA nor PR3-ANCA affected the three poor outcomes. According to the new criteria, 12 (6.7%) and 21 (11.8%) patients were classified as having granulomatosis with polyangiitis (GPA) and microscopic polyangiitis (MPA), respectively. Conclusions: SARS-CoV-2 infection may increase the rate of ANCA positivity. Although it might not affect poor outcomes, it might contribute to the classification of GPA and MPA despite uncertain clinical significance.

## 1. Introduction

Severe acute respiratory syndrome coronavirus 2 (SARS-CoV-2), a novel coronavirus belonging to the genus Betacoronavirus in the family Coronaviridae, was first reported in Wuhan, China in December 2019 [1]. The severity of the Coronavirus Disease 2019 (COVID-19) caused by SARS-CoV-2 infection ranges from mild to severe, with 554 million infected patients and 6.3 million deaths as of the end of February 2022 [2]. Moreover, COVID-19 is well-known for its broad spectrum of immune-related phenotypes similar to those seen in autoimmune or inflammatory diseases [3]. COVID-19 has been shown to provoke a surge in proinflammatory cytokines, including tumour necrosis factor-alpha and interleukin-6, which in turn may switch on the dysregulation of immune responses to the virus [4]. Furthermore, evidence has gradually accumulated that COVID-19 may induce systemic inflammatory manifestations (such as multisystem inflammatory syndrome (MIS-C), haemophagocytic syndromes, and systemic vasculitis) [5] and autoimmune diseases/syndromes (such as immune thrombocytopenic purpura, Guillain-Barré syndrome, and anti-phospholipid antibody associated thrombosis) [6,7]. In addition to increased levels of inflammatory cytokines, the vulnerability of endothelial cells to viral binding and injury owing to their abundant expression of the angiotensin-converting enzyme 2 receptor is another mechanism underlying the multi-organ involvement of inflammation [8]. In patients infected with SARS-CoV-2, neutrophils and mononuclear cells can be recruited to endothelial cells along with viral inclusion structures, which can accelerate inflammation [9]. Furthermore, it has been demonstrated that SARS-CoV-2 infection can contribute to an increase in the formation of neutrophil extracellular traps (NETs), which may cause injury and dysfunction of endothelial cells and further augment apoptotic cell death [10].

Antineutrophil cytoplasmic antibody (ANCA)-associated vasculitis (AAV) is a small vessel vasculitis characterised by necrotising vasculitis with few or no immune deposits, primarily in capillaries, venules, and arterioles and occasionally in arteries. AAV can be divided into three subtypes according to clinical, laboratory, radiologic, and histopathologic features: microscopic polyangiitis (MPA), granulomatosis with polyangiitis (GPA), and eosinophilic GPA (EGPA) [11,12]. Furthermore, a new method for classifying AAV into three subtypes according to autoantigens targeted by ANCAs, namely myeloperoxidase (MPO)-ANCA vasculitis, proteinase 3 (PR3)-ANCA vasculitis, and ANCA-negative vasculitis, has also been proposed [13]. To date, there have been several case reports examining AAV occurrences after COVID-19, indicating the potential of COVID-19 to trigger AAV [14,15]. The link between the pathophysiology of AAV and that of SARS-CoV-2 can be speculated based on the following findings: (i) infection is a well-known environmental risk factor for ANCA production [16]; (ii) SARS-CoV-2 infection may be closely associated with the dysregulation of normal immunity owing to the formation of NETs, which is also a major pathophysiological mechanism of AAV [10,17]; and (iii) excessive NET formation might be involved in AAV occurrence by producing ANCAs, which can accelerate ANCA-mediated neutrophil activation and exacerbate the secretion of proinflammatory cytokines, reactive oxygen species, and granular lytic enzymes, subsequently causing vascular injuries and extravascular immune cell infiltrates [18,19,20,21].

Consistent with this notion, a recently published study has revealed a high detection rate of ANCA positivity in patients with SARS-CoV-2 infection using an indirect immunofluorescence assay for perinuclear (P)-ANCA and cytoplasmic (C)-ANCA [22]. However, studies investigating the detection rate of ANCA positivity using immunoassay for MPO-ANCA and PR3-ANCA or evaluating the clinical significance of ANCA positivity in patients infected with SARS-CoV-2 have not been reported yet. Hence, the objective of this study was to investigate the detection rate of ANCA positivity in patients infected with SARS-CoV-2 requiring hospitalisation and clinical significance of ANCA positivity. Furthermore, this study applied the new classification criteria for AAV proposed by both the American College of Rheumatology (ACR) and the European Alliance of Associations for Rheumatology (EULAR) in 2022 (the 2022 ACR/EULAR criteria) for patients with ANCA positivity and investigated whether they could be classified as having AAV.

## 2. Patients and Methods

### 2.1. Study Subjects

This study was conducted at a large tertiary-care teaching hospital in Seoul, Korea from June 2020 to October 2021. The inclusion criteria were as follows: (i) patients who were over the age of 19; (ii) patients infected with SARS-CoV-2, which was confirmed using a method of reverse transcription-polymerase chain reaction; (iii) patients who were admitted for COVID-19; (iv) patients whose blood samples were collected between 14 and 30 days after the onset of SARS-CoV-2 infection-related symptoms; (v) patients who had never been diagnosed with AAV prior to SARS-CoV-2 infection; (vi) patients who were not treated with convalescent plasma; and (vii) patients who did not receive intravenous immunoglobulin within the previous six months prior to blood sampling. We subsequently selected 178 patients infected with SARS-CoV-2 based on the inclusion criteria and included them in this study. This study was approved by the Institutional Review Board (IRB) of Severance Hospital (4-2020-0076). It was conducted in accordance with the Declaration of Helsinki. Written informed consent was obtained from all patients at the time of blood sampling. The IRB waived the need for additional written informed consent if the informed consent had been previously obtained during blood sampling.

### 2.2. Clinical Data

Data on age and sex were collected as demographic data. Numbers of patients with MPO-ANCA and PR3-ANCA were counted. The cumulative dose of glucocorticoids equivalent to methylprednisolone and the usage duration were assessed. The duration of glucocorticoid use was defined as the period from the first administration to the day of blood sampling. Mortality, mechanical ventilator care, and severe infection were assessed as poor outcomes. Mortality was defined as death due to COVID-19 alone. A high-flow nasal cannula (HFNC) was applied when a peripheral oxygen saturation ≤94% was confirmed despite supplying oxygen through a nasal prong. Mechanical ventilator care was initiated when HFNC was insufficient to maintain peripheral oxygen saturation. Severe infection was defined as a medical condition that required HFNC and/or mechanical ventilator care. The follow-up period based on mortality was defined as the period from diagnosis to death for deceased patients and from diagnosis to the last visit for surviving patients. The follow-up period based on mechanical ventilator care was defined as the period from diagnosis to the initiation of mechanical ventilator care for patients receiving ventilator care and the period from diagnosis to the last visit for those not receiving it. The follow-up period based on severe infection was defined similar to mechanical ventilator care if mechanical ventilator care was changed to severe infection.

### 2.3. Blood Samples

Whole blood samples were collected from study subjects between 14 and 30 days after symptom onset. Sera were isolated from whole blood samples and stored at −70 °C on the day of blood sampling.

### 2.4. ANCA Measurement

MPO-ANCA and PR3-ANCA levels in stored sera were detected using enzyme-linked immunosorbent assay (ELISA) kits (EuroImmun, Lübeck, Germany) according to the manufacturer’s instructions.

### 2.5. Application of the New Classification Criteria for AAV

The 2022 ACR/EULAR classification criteria for the three subtypes of AAV were applied only to patients who had MPO-ANCA or PR3-ANCA among study subjects [23,24].

### 2.6. Statistical Analysis

Statistical analysis was performed using Prism 9 software (GraphPad, San Diego, CA, USA) and IBM SPSS Statistics for Windows, version 26 (IBM Corp., Armonk, NY, USA). Significant differences between two categorical variables were analysed using the Chi-square and Fisher’s exact tests. Significant differences between two continuous variables were compared using the Mann–Whitney U test. Cumulative survival rates between two groups were compared using the Kaplan–Meier survival analysis with the log-rank test. The statistical significance level was set at *p* < 0.05.

## 3. Results

### 3.1. Characteristics of Patients Infected with SARS-CoV-2

The median age of patients was 60.5 years. Of all patients, 62.4% were men. The detection rate of ANCA in patients infected with SARS-CoV-2 was 18.5%. MPO-ANCA and PR3-ANCA were detected in 22 (12.4%) and 14 (7.9%) patients, respectively.

Three patients had both MPO-ANCA and PR3-ANCA. Median cumulative doses of glucocorticoids and usage durations were 656.4 mg and 13.0 days, respectively. Of 178 patients, 22 (12.4%) died of COVID-19 during a median follow-up period of 166.0 days. In addition, 54 (30.3%) required mechanical ventilator care during a median follow-up period of 71.5 days, and 134 (75.3%) patients experienced severe infection during a median follow-up of 9.0 days. The median lag time from symptom onset to blood sampling was 21.0 days (Table 1).

### 3.2. Comparison of Cumulative Survival Rates

**Figure 1:**
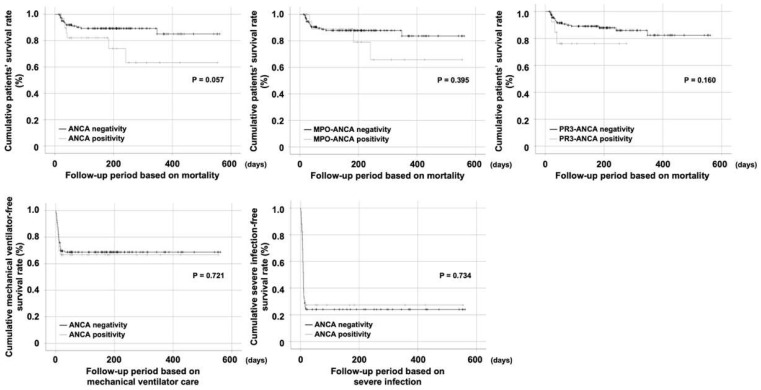
Comparison of cumulative survival rates. Neither ANCA positivity nor ANCA subtype (MPO-ANCA and PR3-ANCA) positivity had a significant influence on poor outcomes of SARS-CoV-2. ANCA: antineutrophil cytoplasmic antibody; MPO: myeloperoxidase; PR3: proteinase 3; SARS-CoV-2: severe acute respiratory syndrome coronavirus 2.

Patients with ANCA positivity exhibited a lower cumulative survival rate than those without. However, the difference between the two was not statistically significant (*p* = 0.057). Cumulative survival rates of patients according to MPO-ANCA positivity or PR3-ANCA positivity did not differ significantly either. Moreover, ANCA positivity did not affect the cumulative mechanical ventilator-free survival rate or severe infection-free survival rate in patients infected with SARS-CoV-2 (Figure 1). We also compared cumulative survival rates of patients with MPO-ANCA positivity and patients with PR3-ANCA positivity. Although the survival curve showed a trend of early death in patients with PR3-ANCA positivity, their survival rates were not significantly different (Figure S1).

### 3.3. Application of the New Classification Criteria for AAV

According to the 2022 ACR/EULAR classification criteria for GPA and MPA [24,25], of 33 patients infected by SARS-CoV-2 with positive detection of ANCAs, 12 (6.7%) and 21 (11.8%) patients were classified as having GPA and MPA, respectively. None of these patients fulfilled the 2022 ACR/EULAR classification criteria for EGPA (Table 2) [23]. Although most ANCA-positive patients met the classification criteria, the diagnosis was not sufficiently reliable since AAV could be mimicked by various infectious diseases such as SARS-CoV-2 infection.

### 3.4. Classification of Each Patient Based on Items of the 2022 ACR/EULAR Criteria Met by at Least One Patient

Items fulfilled by patients in this study are shown in Table 3. Per GPA and MPA, if a patient obtained a total score of ≥5, the patient could be classified as having GPA or MPA. Of the 33 patients who were classified as having GPA or MPA, one was classified as having GPA and MPA simultaneously. Patient 12 was classified as having GPA because of PR3-ANCA positivity (+5 points), MPO-ANCA positivity (−1 point), and paranasal sinusitis (+1 point), with a total score of 5. The patient was also classified as having MPA because of PR3-ANCA positivity (−1 point) and MPO-ANCA positivity (6 points), with a total score of 5. The other two patients (patients 1 and 28) with both MPO-ANCA and PR3-ANCA were classified as having only MPA. Conversely, one patient could not be classified as having either subtype of AAV. Although patient 16 had MPO-ANCA positivity (6 points), the patient was not classified as having MPA due to a serum eosinophil count ≥1000/μL (−4 points), resulting in a total score of only 2. So far, no patient has been treated for AAV. As listed in Table 3, there were a few patients with pulmonary manifestations or sinusitis. However, no patient had biopsy-proven pauci-immune glomerulonephritis.

## 4. Discussion

In this study, we investigated the detection rate and clinical significance of ANCA positivity in patients infected by SARS-CoV-2. Our results indicated that the detection rate of ANCA positivity increased by up to 18.5. However, neither MPO-ANCA nor PR3-ANCA affected the three poor outcomes (cumulative survival rate, mechanical ventilator-free survival rate, and severe infection-free survival rate). It was also revealed that ANCA positivity contributed to the classification of AAV in 97.5% of patients positive for ANCA.

We looked at the incidence rate of ANCA positivity in individuals without AAV to see if the incidence rate of ANCA positivity of 18.5% was significantly higher than that in the general population. Since there are few recent reports on the incidence rate of ANCA positivity in the general population with possible ethnic differences, we referred to two previous studies conducted in Korea and Japan. A previous study screened results of ANCA tests at a single Korean centre for 26,499 individuals who were classified as not having AAV and found that ANCA was detected in 173 individuals, indicating that the incidence rate of ANCA was 0.7% [26]. Another previous study performed ANCA tests for 1,204 individuals at a single Japanese centre and detected MPO-ANCA and/or PR3-ANCA in 11 of them, indicating that the incidence rate of ANCA positivity was 0.9% [27]. Based on these previous results, we concluded that the incidence rate of ANCA in patients infected with SARS-CoV-2 was remarkably increased.

In the proinflammatory environment of a cytokine storm that can be triggered by SARS-CoV-2 [28], MPO-ANCA and PR3-ANCA can be produced and be present in the peripheral circulating blood [16,29,30,31]. A previous study has revealed that ANCAs are detected in 6.9%, 2.3%, 1.2%, and 0.6% of patients infected with varicella-zoster virus, herpes simplex virus, cytomegalovirus, and respiratory syncytial virus, respectively [26]. Another case report has provided information on the association between Epstein–Barr virus and MPO-ANCA positivity [32]. In contrast, it has been reported that SARS-CoV-2 may participate in the production of autoantibodies in healthy individuals [33]. Furthermore, it could initiate and accelerate the cytokine storm [4,34]. Therefore, it can be reasonably concluded that SARS-CoV-2, like other viruses, may activate autoimmunity and accelerate ANCA production through diverse immune cells activated by the cytokine storm.

In this study, ANCA positivity had little influence on poor outcomes of SARS-CoV-2, which might be due to a lag time between the two following different phases of AAV pathogenesis: a neutrophil priming phase and a neutrophil activation phase. In the neutrophil priming phase, external stimuli processed by APCs can activate TH17 cells and increase IL-17 production, which can subsequently activate macrophages and drive them to secrete plenty of various pro-inflammatory cytokines. In such microenvironments, cytoplasmic MPO and PR3 in primed neutrophils can move to the cell surface and become released by lysozymes. Secreted MPO and PR3 are processed by APCs to activate autoreactive T and B cells, leading to the production of MPO-ANCA and PR3-ANCA [28]. During this phase, circulating ANCA positivity may occur. However, it cannot be said that AAV will occur. In terms of the neutrophil activation phase, once primed neutrophils form dimers or multimers with circulating ANCA, ANCA-mediated activation of neutrophils can be initiated, which in turn may migrate into vessel-adjacent tissues, secrete various granular enzymes and oxygen radicals, and finally induce tissue damage or form granulomas [28,35], leading to the final development of AAV. Given the lag time, we can conclude that ANCA positivity does not imply the occurrence or progression of AAV directly. In addition, given the difficulty in distinguishing between pathogenic ANCA and non-pathogenic ANCA (natural ANCA) [36], we can also conclude that ANCA positivity itself may not warrant its effect on poor outcomes of SARS-CoV-2, although natural ANCAs might be transformed into pathogenic ANCAs through impairment of T cell and B cell suppression [36].

Unexpectedly, when the 2022 ACR/EULAR classification criteria for AAV were applied to 33 patients with ANCAs, 32 of them could be classified as having GPA or MPA in this study. The new ACR/EULAR classification criteria for GPA and MPA are composed of a scoring system. When a total score of 5 is achieved, GPA and MPA could be classified based on them. The biggest reason that most patients with ANCA could be classified as GPA and MPA is that the weighted proportion of MPO-ANCA and PR3-ANCA in the new diagnostic classification criteria has significantly increased compared to the previous criteria. In the classification of GPA and MPA, 5 points are assigned to PR3-ANCA and MPO-ANCA, respectively, whereas, conversely, only a negative point is assigned to MPO-ANCA and PR3-ANCA [24,25]. In addition, the lack of distinct clinical, laboratory, radiologic, and histologic items to which negative points are assigned might also have contributed to the high classification rate in this study. However, the classification of 32 patients as having GPA or MPA could not be confirmed because the 2022 ACR/EULAR classification criteria required two entry requirements. First, there should be evidence of small or medium vessel vasculitis. Second, other diseases mimicking AAV, such as concomitant malignancies, serious infectious diseases, and other autoimmune diseases, must be excluded. Therefore, it may be controversial to classify patients infected with SARS-CoV-2 as having GPA or MPA who have not been monitored through a sufficiently long follow-up period. Our results suggest that regular and close monitoring should be provided to the 32 patients who met the 2022 ACR/EULAR classification criteria for AAV.

This study has two notable merits. First, we assessed the detection rate of ANCA positivity in patients infected with SARS-CoV-2 requiring hospitalisation using an immunoassay for MPO-ANCA and PR3-ANCA instead of an indirect immunofluorescence assay. Second, we applied the 2022 ACR/EULAR classification criteria for AAV and investigated the proportion of patients classified as having AAV for the first time.

However, this study has several limitations. First, the number of patients was not sufficiently large to apply results of this study to all patients infected with SARS-CoV-2 and represent them. Second, the retrospective study design of this study might have reduced the reliability of results of this study. Third, the follow-up period was not sufficiently long to monitor the development of systemic manifestations of AAV in patients with ANCAs. Fourth, long-term complications or sequelae of COVID-19 were not evaluated in this study. Thus, a prospective, nationwide study with a larger number of patients is needed to validate results of this study and clarify the association between SARS-CoV-2 infection and AAV occurrence.

In conclusion, the detection rate of ANCA positivity in patients infected with SARS-CoV-2 was 18.5%. ANCA positivity made a significant contribution to the classification of AAV. However, ANCA positivity had no significant influence on poor outcomes of COVID-19.

## Figures and Tables

**Table 1:** Characteristics of patients infected with SARS-CoV-2 (*n* = 178).

Variables	Values
**Demographic data**	
Age (years)	65.0 (20.0)
Male sex (N (%))	111 (62.4)
**ANCA positivity (N (%))**	
Any ANCA positivity	33 (18.5)
MPO-ANCA positivity	22 (12.4)
PR3-ANCA positivity	14 (7.9)
Both ANCA positivity	3 (0.2)
ANCA negativity	145 (81.5)
**Glucocorticoid use**	
Cumulative dose equivalent to methylprednisolone (mg)	656.4 (691.5)
Usage duration (days)	13.0 (10.0)
**Clinical outcomes (N (%))**	
Mortality	22 (12.4)
Mechanical ventilator care	54 (30.3)
HFNC	80 (44.9)
Severe infection (Mechanical ventilator care + HFNC)	134 (75.3)
**Follow-up period (days)**	
Follow-up period based on mortality	166.0 (192.0)
Follow-up period based on mechanical ventilator care	71.5 (182.0)
Follow-up period based on severe infection	9.0 (12.0)
**Lag time from symptom onset to blood sampling (days)**	21.0 (7.0)

Values are expressed as median (interquartile range) or number (percentage). SARS-CoV-2: severe acute respiratory syndrome coronavirus 2; ANCA: antineutrophil cytoplasmic antibody; MPO: myeloperoxidase; PR3: proteinase 3; HFNC: high flow nasal cannula.

**Table 2:** Application of 2022 ACR/EULAR classification criteria for GPA, MPA, or EGPA to patients infected by SARS-CoV-2 with positive detection of ANCAs (*n* = 33).

Variables	Number of Patients
At the time of first symptom	
**Clinical criteria**	
Nasal involvement	0
Cartilaginous involvement	0
Conductive or sensorineural hearing loss	0
Obstructive airway disease	3 (9.1)
Nasal polyp	0
Mononeuritis multiplex	0
**Laboratory criteria**	
PR3-ANCA positivity	14 (42.4)
MPO-ANCA positivity	22 (66.7)
Serum eosinophil ≥1000/µL	2 (6.1)
Haematuria	20 (60.6)
**Biopsy criteria**	
Granuloma, granulomatous inflammation, or giant cells	N/A
Pauci-immune glomerulonephritis	N/A
Extravascular eosinophilic-predominant inflammation	N/A
**Imaging criteria**	
Pulmonary nodules, mass, or cavitation on chest imaging	3 (9.1)
Fibrosis or ILD on chest imaging	4 (12.1)
Nasal/paranasal sinusitis or mastoiditis on imaging	6 (18.2)
**Number of patients with total score ≥5 for GPA = 12** **Number of patients with total score ≥5 for MPA = 21** **Number of patients with total score ≥6 for EGPA = 0**	

Values are expressed as number (percentage). ACR: American College of Rheumatology; EULAR: European Alliance of Associations for Rheumatology; GPA: granulomatosis with polyangiitis; MPA: microscopic polyangiitis; EGPA: eosinophilic granulomatosis with polyangiitis; SARS-CoV2: severe acute respiratory syndrome coronavirus 2; ANCA: Antineutrophil cytoplasmic antibody; PR3: proteinase 3; MPO: myeloperoxidase; ILD: interstitial lung disease.

**Table 3:** Classification of each patient infected by SARS-CoV-2 with positive detection of ANCA based on items of the 2022 ACR/EULAR criteria met by at least one patient.

Patients Number	Classification Criteria								Score for GPA	Score for MPA	Score for EGPA
	Obstructive Airway Disease	PR-3 ANCA Positivity	MPO ANCA Positivity	Serum Eosinophil ≥1000/µL	Haematuria	Pulmonary Nodules, Mass, or Cavitation on Chest Imaging	Fibrosis or ILD on Chest Imaging	Nasal/Paranasal Sinusitis or Mastoiditis on Imaging			
1		+	+		+				4	5	−4
2			+						−1	6	0
3			+	+	+	+	+	+	−2	5	4
4			+		+				−1	6	−1
5			+	+	+		+		−1	9	−1
6			+						−1	6	0
7		+			+		+		5	2	−4
8	+		+						−1	6	3
9			+		+				−1	6	−1
10		+			+				5	−1	−4
11		+			+				5	−1	−4
12		+	+		+			+	5	5	−4
13		+							5	−1	−3
14			+		+	+		+	2	6	−1
15		+			+				5	−1	−4
16			+	+				+	−4	2	5
17		+			+				5	−1	−4
18		+							5	−1	−3
19	+		+		+				−1	6	2
20			+						−1	6	0
21		+							5	−1	−3
22			+						−1	6	0
23			+						−1	6	0
24			+		+				−1	6	−1
25		+							5	−1	−3
26			+		+		+		−1	9	−1
27			+			+			1	6	0
28		+	+		+				4	5	−4
29		+			+				5	−1	−4
30			+		+			+	0	6	−1
31		+							5	−1	−3
32	+		+		+				−1	6	2
33			+		+			+	0	6	−1

+: Fulfilled items by each patient; ACR: American College of Rheumatology; EULAR: European Alliance of Associations for Rheumatology; ANCA: Antineutrophil cytoplasmic antibody; GPA: granulomatosis with polyangiitis; MPA: microscopic polyangiitis; EGPA: eosinophilic granulomatosis with polyangiitis; MPO: myeloperoxidase; PR3: proteinase 3, ILD: interstitial lung disease.

## Data Availability

Data underlying this article are available from the corresponding author upon reasonable request.

## Supplementary Materials

The following supporting information can be downloaded at: https://www.mdpi.com/article/10.3390/jcm11144152/s1, Figure S1. Comparison of cumulative survival rates between patients with MPO-ANCA positivity and patients with PR3-ANCA positivity. There were no significant differences in poor outcomes of SARS-CoV-2 between patients with MPO-ANCA and those with PR3-ANCA. MPO: myeloperoxidase; PR3: proteinase 3; ANCA: antineutrophil cytoplasmic antibody; SARS-CoV-2: severe acute respiratory syndrome coronavirus 2.
