# Supplementary material for: Clinical Significance of Antineutrophil Cytoplasmic Antibody Positivity in Patients Infected with SARS-CoV-2
Source: J Clin Med. 2022 Jul 17;11(14):4152. doi: 10.3390/jcm11144152 (PMC9322989; doi:10.3390/jcm11144152)
Supplement: Supplementary file 1 [file jcm-11-04152-s001.zip › jcm-1778607-supplementary.pdf]

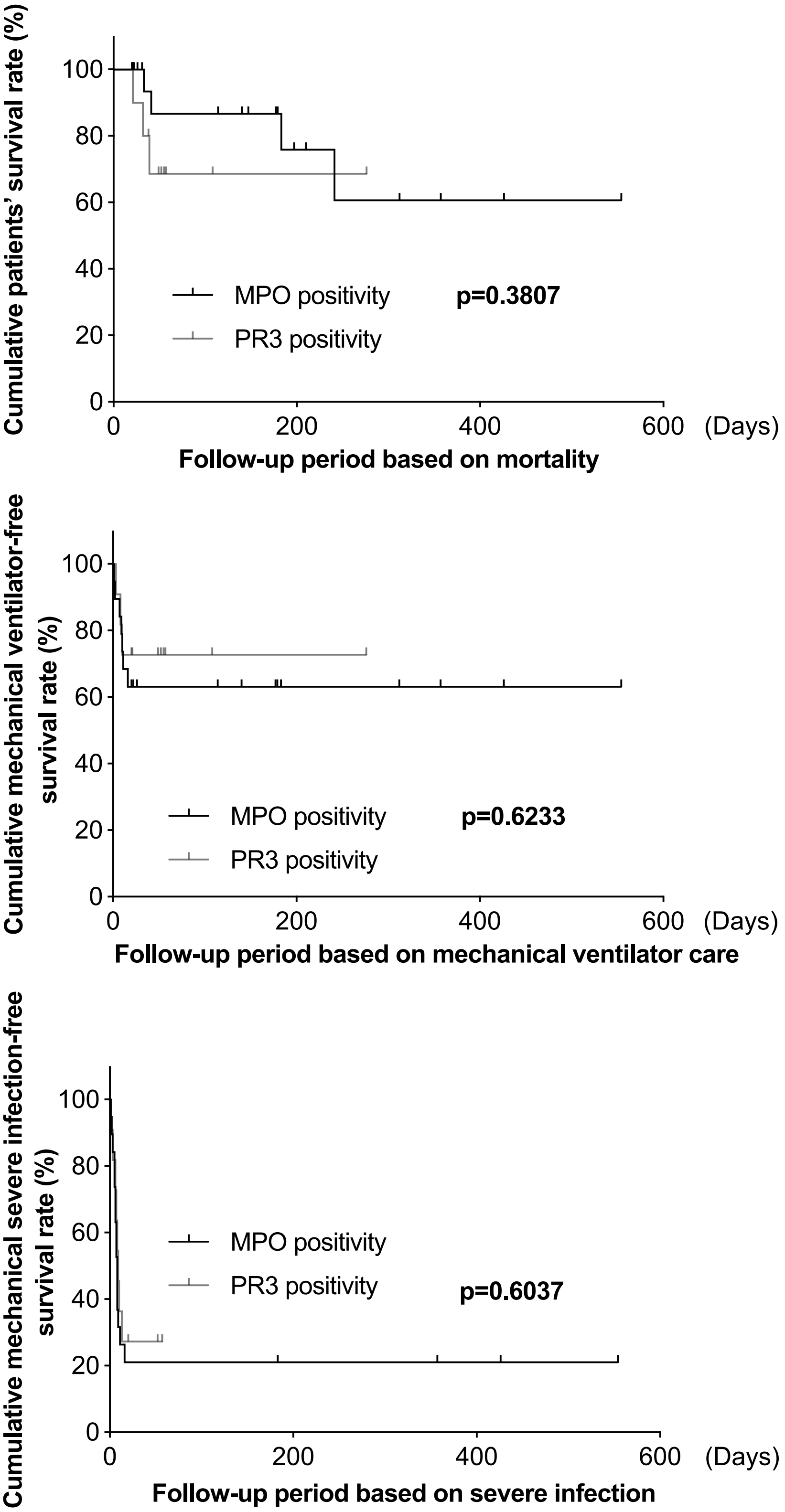

Figure S1. Comparison of cumulative survival rates between patients with MPO-ANCA positivity and patients with PR3-ANCA positivity. There were no significant differences in poor outcomes of SARS-CoV-2 between patients with MPO-ANCA and those with PR3-ANCA.
MPO: myeloperoxidase; PR3: proteinase 3; ANCA: antineutrophil cytoplasmic antibody; SARS-CoV-2: severe acute respiratory syndrome coronavirus 2.
